# Endothelial Exosome Plays a Functional Role during Rickettsial Infection

**DOI:** 10.1128/mBio.00769-21

**Published:** 2021-05-11

**Authors:** Yakun Liu, Changcheng Zhou, Zhengchen Su, Qing Chang, Yuan Qiu, Jiani Bei, Angelo Gaitas, Jie Xiao, Alexandra Drelich, Kamil Khanipov, Yang Jin, Georgiy Golovko, Tais B. Saito, Bin Gong

**Affiliations:** a Department of Pathology, University of Texas Medical Branch, Galveston, Texas, USA; b Department of Mathematics and Statistics, Texas Tech University, Lubbock, Texas, USA; c The Estelle and Daniel Maggin Department of Neurology, Icahn School of Medicine at Mount Sinai, New York, New York, USA; d Department of Pharmacology, University of Texas Medical Branch, Galveston, Texas, USA; e Division of Pulmonary and Critical Care Medicine, Department of Medicine, Boston University Medical Campus, Boston, Massachusetts, USA; University of Arkansas for Medical Sciences; University of Oklahoma Health Sciences Center

**Keywords:** exosome, extracellular vesicle, endothelial cell, endothelial barrier function, spotted fever group rickettsial infection, barrier function, rickettsial infection

## Abstract

Spotted fever group rickettsioses (SFRs) are devastating human infections. Vascular endothelial cells (ECs) are the primary targets of rickettsial infection. Edema resulting from EC barrier dysfunction occurs in the brain and lungs in most cases of lethal SFR, but the underlying mechanisms remain unclear. The aim of the study was to explore the potential role of *Rickettsia*-infected, EC-derived exosomes (Exos) during infection. Using size exclusion chromatography (SEC), we purified Exos from conditioned, filtered, bacterium-free media collected from Rickettsia parkeri-infected human umbilical vein ECs (HUVECs) (*R*-ECExos) and plasma of Rickettsia australis- or *R. parkeri*-infected mice (*R*-plsExos). We observed that rickettsial infection increased the release of heterogeneous plsExos, but endothelial exosomal size, morphology, and production were not significantly altered following infection. Compared to normal plsExos and ECExos, both *R*-plsExos and *R*-ECExos induced dysfunction of recipient normal brain microvascular ECs (BMECs). The effect of *R*-plsExos on mouse recipient BMEC barrier function is dose dependent. The effect of *R*-ECExos on human recipient BMEC barrier function is dependent on the exosomal RNA cargo. Next-generation sequencing analysis and stem-loop quantitative reverse transcription-PCR (RT-qPCR) validation revealed that rickettsial infection triggered the selective enrichment of endothelial exosomal mir-23a and mir-30b, which potentially target the endothelial barrier. To our knowledge, this is the first report on the functional role of extracellular vesicles following infection by obligately intracellular bacteria.

## INTRODUCTION

Spotted fever group (SFG) rickettsioses (SFRs) are devastating human infections (1). A licensed vaccine is not available. It is forecasted that increased ambient temperatures under conditions of global climate change will lead to more widespread distribution of rickettsioses (2). These arthropod-borne diseases are caused by obligately intracellular bacteria of the genus *Rickettsia*, including Rickettsia rickettsii (3, 4) and *R. parkeri* (5–7), which cause Rocky Mountain spotted fever and *R. parkeri* rickettsiosis (8), respectively, in the United States and Latin America; R. conorii, the causative agent of Mediterranean spotted fever endemic to southern Europe, North Africa, and India (9); and *R. australis*, which causes Queensland tick typhus in Australia (10). Vascular endothelial cells (ECs) are the primary targets of infection, and EC tropism plays a central role during pathogenesis (1, 3, 11). Edema resulting from EC barrier dysfunction occurs in the brain and lungs in most cases of lethal SFR (12). Typically, rickettsial infection is controlled by appropriate broad-spectrum antibiotic therapy if diagnosed early (3, 4). However, rickettsial infections can cause nonspecific signs and symptoms, rendering early clinical diagnosis difficult (13, 14). Untreated or misdiagnosed rickettsial infections are frequently associated with severe morbidity and mortality (1, 4, 15–17). A fatality rate as high as 32% has been reported for hospitalized patients with Mediterranean spotted fever (17). Although doxycycline is the antibiotic of choice for rickettsial infections, it only stops bacteria from reproducing and does not kill the rickettsiae. Comprehensive understanding of rickettsial pathogenesis is urgently needed for the development of novel therapeutics (7, 16, 18–22).

Eukaryotic cell-to-cell communication is critical for maintaining homeostasis and responding quickly to environmental stimuli (23–51). Besides direct intercellular contact, this communication is often mediated by soluble factors that can convey signals to a large repertoire of responding cells, either locally or remotely. Extracellular vesicles (EVs) transfer functional mediators to neighboring and distant recipient cells (33). EVs are broadly classified into two categories, exosomes (Exos) (50 to 150 nm) and microvesicles (100 to 1,000 nm), owing to their endocytic or plasma membrane origin (38, 52–66). Exos and microvesicles are also termed small and large EVs, respectively (55). Exo biogenesis begins with the formation of intraluminal vesicles, the intracellular precursors of Exos, after the inward budding of the membranes of late endosomes (37, 54). Intraluminal vesicles are internalized into a multivesicular body, which transits toward and fuses with the plasma membrane, before releasing intraluminal vesicles into the extracellular environment as Exos (53). An Exo contains many types of biomolecules, including proteins, nucleic acids, and lipids (67). Once bound to the plasma membrane of the recipient cell, Exos can induce functional responses by multiple mechanisms, e.g., activating receptors on recipient cells or releasing their bioactive cargos after internalization (67, 68). In infectious biology, EVs from infected donor cells contain cargos that are associated with the virulence of the pathogen or the activation of host self-defense mechanisms (33–38, 69–71). EVs released from macrophages infected by intracellular bacteria, such as Mycobacterium tuberculosis and Salmonella enterica serovar Typhimurium, have been shown to stimulate a proinflammatory response in noninfected macrophages in a Toll-like receptor-dependent manner (70). Unfortunately, the role(s) of EVs in the pathogenesis of obligately intracellular bacterial infections remains unknown.

Although small noncoding RNA (sncRNA) species (<150 nucleotides) are relatively stable compared with other RNA molecules, they remain vulnerable to RNase-mediated digestion (72). The discovery of extracellular sncRNAs in the blood, despite the abundant presence of RNases, led to the proposal of a scenario in which sncRNAs are encapsulated in EVs (55, 72–74) or form circulating ribonucleoproteins (75, 76). Extracellular RNAs are enriched in sncRNAs (77). A growing number of reports have established that many, if not all, of the effects of EVs are mediated by microRNA (52, 55–60, 63) or tRNA fragment (61, 78) cargos, which remain functional to regulate cellular behaviors of the recipient cells (79). Recent studies provide emerging evidence that microRNAs are selectively sorted into EVs independently of their cellular levels (52, 55–62). We reported that R. conorii infection induces significant upregulation of specific tRNA-derived RNA fragments in host cells, but no global changes of microRNAs in perfusion-rinsed mouse lung tissues were observed (80). Information regarding the potential role of extracellular RNAs during rickettsial infections is still lacking.

The aim of this study was to explore the potential role of rickettsia-infected, EC-derived Exos following infection. Using size exclusion chromatography (SEC), we purified Exos from conditioned, filtered, bacterium-free media collected from *R. parkeri*-infected human umbilical vein ECs (HUVECs) (*R*-ECExos) and plasma of *R. australis*- or *R. parkeri*-infected mice (*R*-plsExos). We observed that compared to noninfectious normal mouse plsExos and normal HUVEC-derived Exos, both *R*-plsExos and *R*-ECExos induced dysfunction of normal brain microvascular ECs (BMECs). The effect of *R*-plsExos on mouse recipient BMEC barrier function is dose dependent. The effect of *R*-ECExos on human recipient BMEC barrier function is dependent upon exosomal RNA cargos. Saponin-assisted active exosomal permeabilization pretreatment (81–83) of *R*-ECExos with RNase mitigated the effect of *R*-ECExos on human recipient BMEC barrier function. Next-generation sequencing analysis and stem-loop quantitative reverse transcription-PCR (RT-qPCR) validation revealed that *R. parkeri* infection triggered the selective enrichment of endothelial exosomal mir-23a and mir-30b, which potentially target the endothelial barrier.

## RESULTS

### Quality assessment of bacterium-free *R*-plsExos and media of *R*-ECExos.

Using SEC, we isolated small EVs (50 to 150 nm) from rickettsia-infected mouse plasma and HUVECs from culture media; both were passed through two 0.2-μm filters. Quantitative real-time PCR validated that no rickettsial DNA copies were detected in either *R*-plsExos isolated from *R. australis*- or *R. parkeri-*infected mice infected with 2 50% lethal doses (LD_50_) of bacteria (80, 84–87) on day 4 postinfection (p.i.) or the *R*-ECExos that were purified 72 h p.i. from *R. parkeri*-infected HUVECs (6) using a multiplicity of infection (MOI) of 10 (Fig. S1).

10.1128/mBio.00769-21.2 FIG S1 Quantities of rickettsiae in plasma-derived Exos (plsExos) (*n* = 5/group) and HUVEC culture media-derived Exos (ECExos) (*n* = 3/group), determined by quantitative real-time PCR. Data are presented as means ± standard errors. Statistical significance was determined using one-way analysis of variance. **, *P < *0.01. Download FIG S1, JPG file, 0.1 MB. Copyright © 2021 Liu et al. 2021 Liu et al. https://creativecommons.org/licenses/by/4.0/ This content is distributed under the terms of the Creative Commons Attribution 4.0 International license.

**FIG 1:**
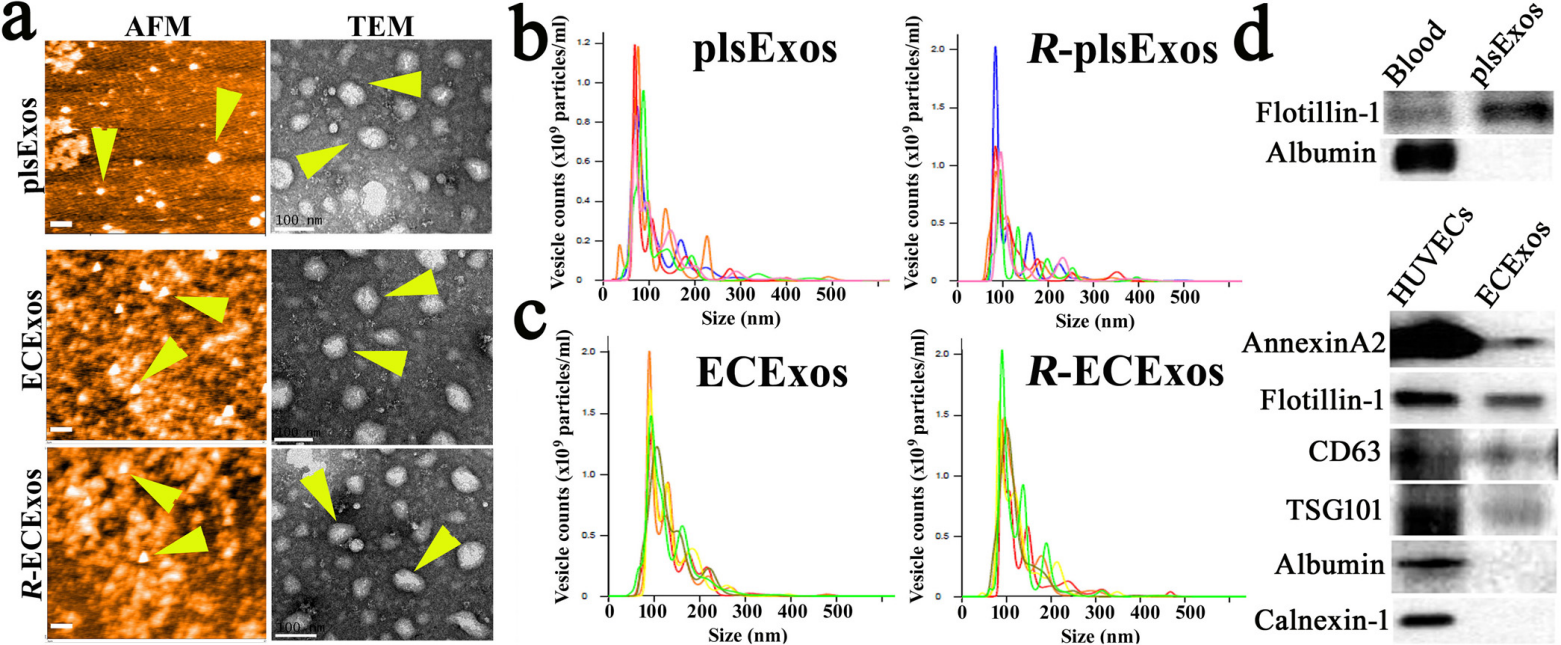
Characterization of plsExos and ECExos after SEC isolation. (a) plsExos and ECExos morphologies were verified using atomic force microscopy (AFM) (left; scale bars, 200 nm) and transmission electronic microscopy (TEM) (right; scale bars, 100 nm). (b and c) The vesicle size distribution of isolated EVs was analyzed using nanoparticle tracking analysis (NTA) (*n* = 5 per group). (d) Expressions of indicated protein markers in 100 μg of proteins of plsExos (upper portion) and ECExos (lower portion) were examined using Western immunoblotting.

Sizes and morphologies of isolated EVs from mouse plasma and EC culture media, respectively, were initially evaluated using transmission electron microscopy (TEM) (52, 88) or atomic force microscopy (AFM) (89). The images captured using TEM and AFM show particles with typical exosomal morphology (arrowheads in Fig. 1a), as published previously (52, 88, 90). Using nanoparticle tracking analysis (NTA), the size distribution of isolated EVs was also confirmed to be in the range of 50 to 150 nm, which is the expected size of Exos (Fig. 1b and c). We also verified the purity of isolated Exos using Western immunoblotting to detect traditional exosomal markers as shown in Fig. 1d (64, 73, 90).

These data demonstrate that purified EVs from rickettsia-infected mouse plasma or culture media used in these studies were free of bacteria or bacterial DNA, were intact and did not aggregate, and fell within the expected size range of Exos.

### Exos are differentially induced and detected in mouse plasma and EC culture media in response to rickettsial infection.

**FIG 2:**
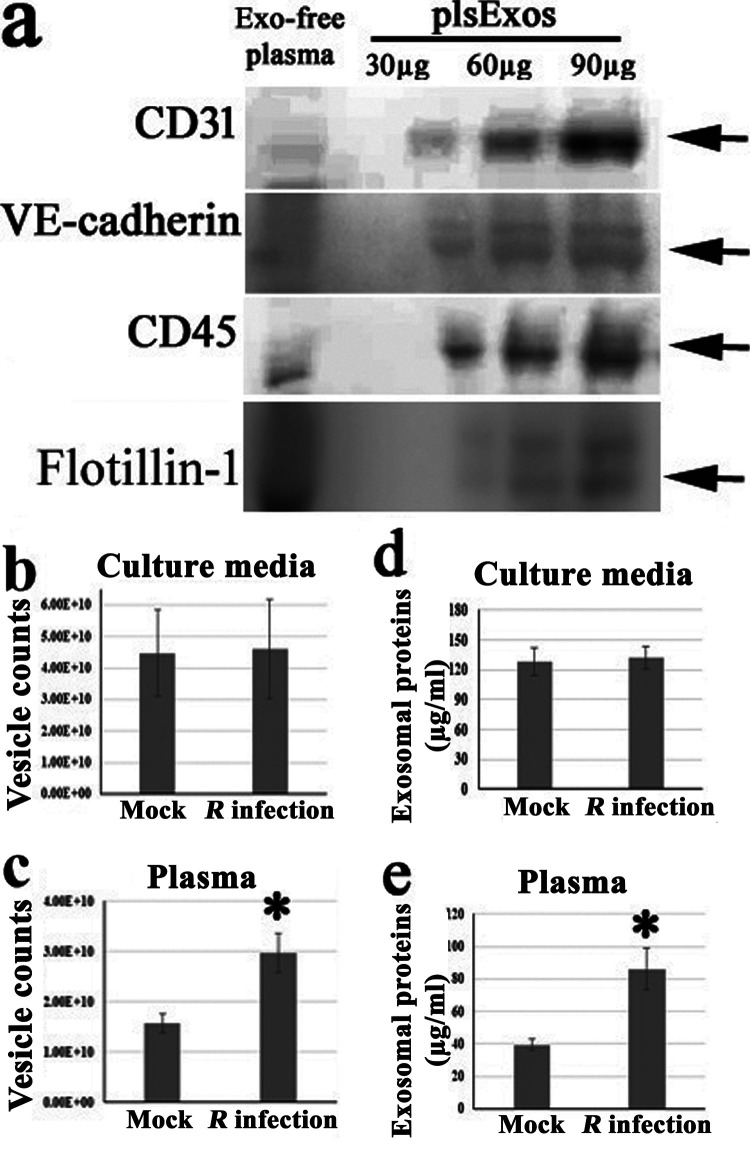
Exos are differentially induced and detected in mouse plasma and EC culture media in response to rickettsial infection. (a) Expression of indicated protein markers (i.e., 30, 60, and 90 μg of plsExos proteins) was examined using Western immunoblotting. (b and c) The concentration of plsExos and ECExos was analyzed using NTA (*n* = 5 per group). (d and e) The concentration of exosomal total protein was determined using the micro-bicinchoninic acid (microBCA) protein assay (*n* = 5 per group). Statistical significance was determined using Student’s *t* test. *, *P* < 0.05.

Exos from circulating blood have been identified as being heterogeneous and derived from multiple cell types, including ECs. Using Western immunoblotting, we detected Exo and EC markers (CD31 and VE-cadherin [91, 92]) in mouse plsExos, as well as markers of other cells (CD45) (Fig. 2a), suggesting that the mouse plsExos used in these studies were derived from different types of cells, including ECs.

Exosomal particle counts were measured using NTA and showed that similar numbers of endothelial Exos were produced by mock and *R. parkeri* infection groups *in vitro* (Fig. 2b). However, the number of mouse *R*-plsExos was upregulated on day 4 p.i. (*P* = 0.02) *in vivo* (Fig. 2c) in the model of *R. australis* infection. Exos were also assessed using exosomal total protein content (Fig. 2d and e) (88). The generation of *R*-plsExos was significantly upregulated on day 4 p.i. in both *in vivo* models of *R. australis* (*P* = 0.016) (Fig. 2e) and *R. parkeri* (*P* = 0.014) (Fig. S2a) infections. No difference in exosomal total protein content was observed between ECExos and *R*-ECExos in the *R. parkeri* infection model (Fig. 2d). Furthermore, we also compared the morphology of EC-derived Exos using TEM and AFM, which demonstrated no significant differences between normal ECExos and *R*-ECExos (Fig. 1a).

10.1128/mBio.00769-21.3 FIG S2 (a) Quantities of plsExos on day 4 postinfection from mock-treated mice or mice infected with 2 LD_50_ of *R. parkeri* given by the intravenous route were assessed using the total protein concentration of plsExos (*n* = 5 per group). Statistical significance was determined using Student’s *t* test. (b) The TEER value of normal mouse recipient BMECs was measured after treatment with normal plsExos (mock) or *R*-plsExos (from an *R. parkeri*-infected mouse) at 400, 2,000, or 8,000 particles per cell for 72 h. Statistical significance was determined using one-way analysis of variance. *, *P* < 0.05. Download FIG S2, JPG file, 0.08 MB. Copyright © 2021 Liu et al. 2021 Liu et al. https://creativecommons.org/licenses/by/4.0/ This content is distributed under the terms of the Creative Commons Attribution 4.0 International license.

Collectively, these data suggest that *R. australis* and *R. parkeri* infections increase heterogeneous plsExo release. However, endothelial Exo size, morphology, and production were not significantly altered after *R. parkeri* infection *in vitro*.

### Recipient cells efficiently take up Exos.

**FIG 3:**
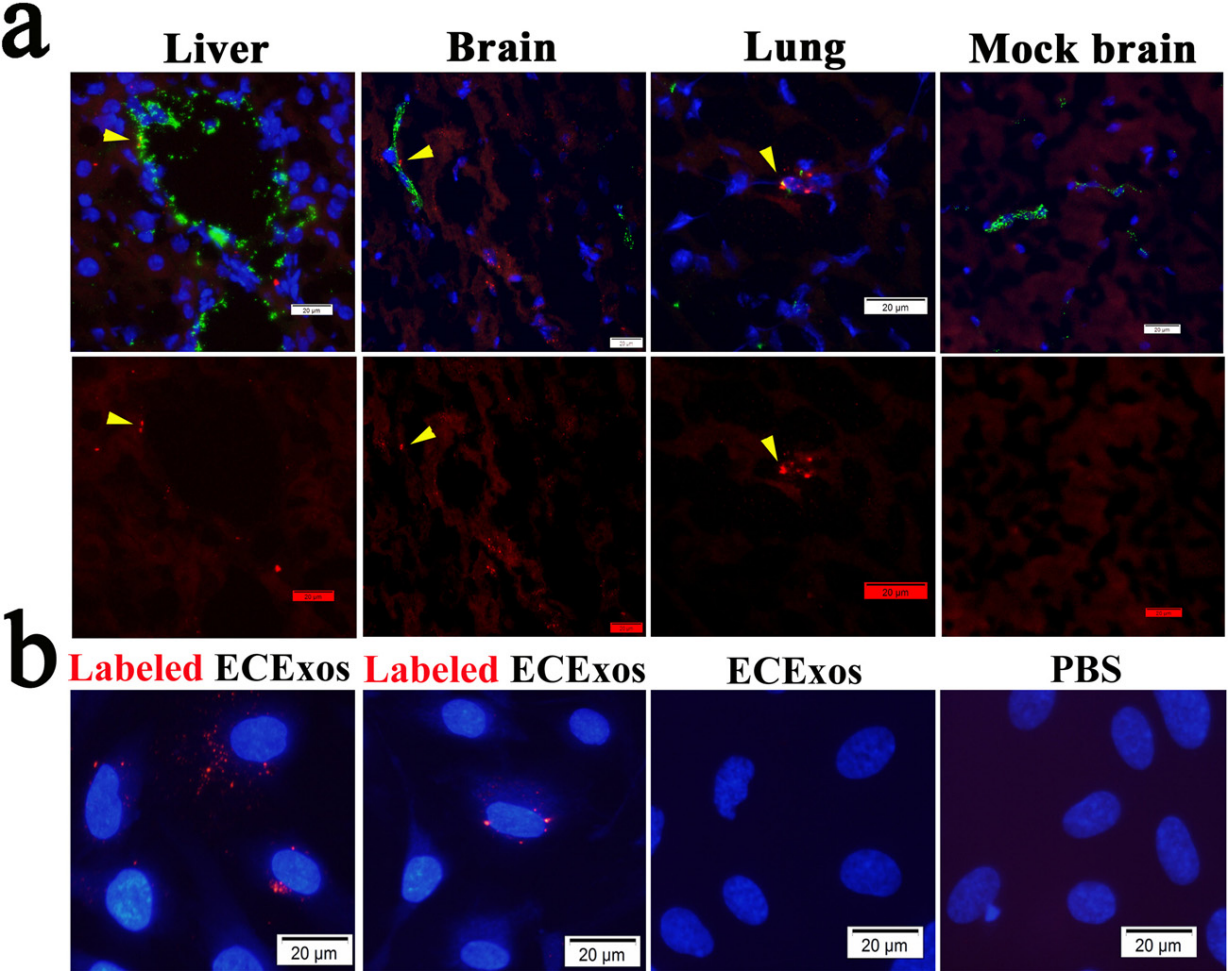
Recipient cells take up Exos. (a) Purified plsExos (5 × 10^10^ particles in 50 μl PBS) labeled with PKH26 were administered to normal C57BL/6J mice intravenously (*n* = 3). After 4 h, organs were dissected for frozen sectioning after euthanasia and perfusion via the right ventricle. Representative immunofluorescent staining of ECs from liver, brain, and lung using an antibody against CD31 (an EC marker) is shown. Normal rabbit serum was used as an antibody during immunofluorescent staining (Fig. S3). The nuclei were stained with 4′,6-diamidino-2-phenylindole (DAPI). Cells with red fluorescence indicate the uptake of PKH26 labeled Exos. Scale bars, 20 μm. (b) Purified ECExos were labeled with PKH26 (red) and added to the culture medium of human BMECs (2,000 particles per cell) as indicated. Pictures were taken using fluorescence microscopy after 2 h of ECExo incubation. Scale bars, 20 μm.

ECs are directly exposed to circulating substances and Exos, which are abundant in blood and are taken up by ECs (93, 94). To confirm that ECs take up Exos *in vivo*, we intravenously delivered fluorescent PKH26-prelabeled normal plsExos (1 × 10^11^ particles per mouse in 100 μl of phosphate-buffered saline [PBS]) to normal mice as described previously (90). As shown in Fig. 3a, colocalization between PKH26 (red) and CD31 (green, a marker of EC lineage) (arrowheads) were identified in multiple organs in mice, which were extensively perfused with PBS at 6 h postinjection, prior to fixation. These data suggest that plsExos directly interact with ECs *in vivo*.

10.1128/mBio.00769-21.4 FIG S3 Normal rabbit serum was used as a control antibody during immunofluorescence (IF) staining of a mouse lung tissue sample treated in same way. Scale bar indicates 20 μm. Download FIG S3, JPG file, 0.1 MB. Copyright © 2021 Liu et al. 2021 Liu et al. https://creativecommons.org/licenses/by/4.0/ This content is distributed under the terms of the Creative Commons Attribution 4.0 International license.

Next, we examined HUVEC-derived ECExo uptake using normal recipient cells (i.e., human BMECs) *in vitro*. PKH26-prelabeled ECExos and nonlabeled controls were added to the cultured human BMECs. As early as 2 h after incubation, the uptake of PKH26-prelabeled ECExos by BMECs was visualized using fluorescence microscopy (Fig. 3b).

These data suggest that vascular ECs efficiently take up Exos in our models.

### Effect of mouse *R*-plsExos on normal mouse recipient ECs.

**FIG 4:**
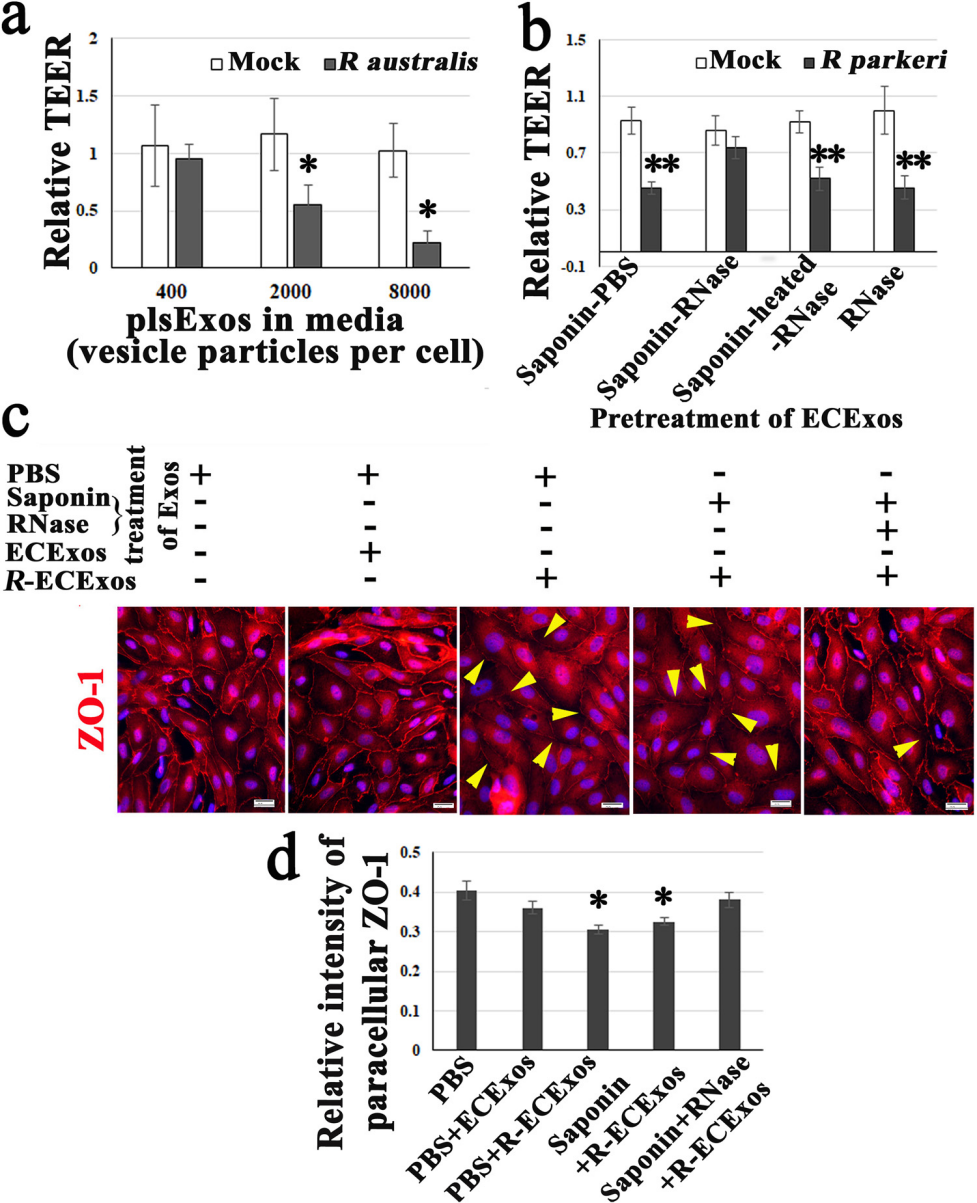
Effect of *R*-plsExos or *R*-ECExos on normal recipient ECs. (a) The transendothelial electrical resistance (TEER) values of normal mouse recipient BMECs were measured after treatment with normal plsExos (mock) or *R*-plsExos at 400, 2,000, or 8,000 Exo particles per cell for 72 h. *, *P* < 0.05. (b) The TEER values of normal human recipient BMECs were measured after a 72-h treatment with normal ECExos (mock) or *R*-ECExos (2,000 Exo particles per cell), which were pretreated with 20 μg/ml of RNase in the presence or absence of 0.1% saponin. **, *P* < 0.01. (c) Immunofluorescence staining of tight junctional protein ZO-1 (red) in normal human recipient BMECs that were treated with different Exos for 72 h. The yellow arrowheads indicate the decreased signals of paracellular ZO-1. Nuclei of human recipient BMECs were counterstained with DAPI (blue). (d) Relative fluorescent intensities of paracellular ZO-1. Normal rabbit serum was used as negative reagent control during immunofluorescent staining (Fig. S4). Scale bars, 20 μm. Statistical significance was determined using one-way analysis of variance. *, *P* < 0.05.

We next sought to evaluate the potential effect of *R*-plsExo on normal recipient ECs during rickettsial infection. Using SEC, *R*-plsExos from a mouse that was intravenously infected with a 2-LD_50_ dose of *R. australis* (86, 87) or *R. parkeri* (6, 95) were isolated. Normal mouse recipient BMECs were treated with normal plsExos or *R*-plsExos at different doses (i.e., 400, 2,000, or 8,000 Exo particles/per cell) for 72 h before measurement of the transendothelial electrical resistance (TEER), an indicator for endothelial paracellular barrier function (96). We found that compared to normal mouse plsExos, mouse *R*-plsExos derived on day 4 p.i. with *R. australis or R. parkeri* reduced the TEER in normal mouse recipient ECs in different dose-dependent manners (Fig. 4a and Fig. S2b).

10.1128/mBio.00769-21.5 FIG S4 Normal rabbit serum was used as a control antibody during IF staining of human BMECs treated in same way. Scale bar indicates 20 μm. Download FIG S4, JPG file, 0.06 MB. Copyright © 2021 Liu et al. 2021 Liu et al. https://creativecommons.org/licenses/by/4.0/ This content is distributed under the terms of the Creative Commons Attribution 4.0 International license.

This evidence suggests that mouse *R. australis*- or *R. parkeri*-plsExos induce dysfunction in normal recipient ECs in a dose-dependent manner.

### Human *R*-ECExos induced dysfunction of normal human recipient ECs in an exosomal RNA-dependent manner.

Endothelial markers were detected in plsExos (Fig. 2a). Given that ECs are the major target cells during rickettsial infection, HUVEC-derived ECExos were used to explore their effect on normal human recipient BMEC function.

It was first found that compared with normal (mock) ECExos (2,000 Exo particles), *R*-ECExos (2,000 Exo particles) reduced TEER in normal human recipient BMECs (Fig. 4b). Furthermore, *R*-ECExos (2,000 Exo particles/cell) weakened the tight junctional protein ZO-1 (arrowheads in Fig. 4c) of normal human recipient BMECs.

Exos contain many types of biomolecules, including proteins and nucleic acids, which contribute to disease pathogenesis (68). Active encapsulation techniques have been widely employed in the field of EV research, showing no significant impairment of exosomal constitution, integrity, or functionality (81–83). To identify the functional exosomal cargos during *R. parkeri* infection, we employed saponin-assisted active permeabilization (81–83) to pretreat exosomal cargos with 20 μg/ml of RNase in the presence of 0.1 mg/ml of saponin. Such pretreatment of *R*-ECExos mitigated the effect on TEER in normal human recipient BMECs compared to RNase in the absence of permeabilization or heat-treated RNase in the presence of saponin (Fig. 4b). Similar pretreatment of *R*-ECExos with RNase in the presence of saponin also mitigated the effect on the tight junctional protein ZO-1 in normal human recipient BMECs (Fig. 4c and d).

We also isolated Exos from HUVEC culture media at 72 h after treatment with heat-inactivated *R. parkeri* (MOI, 10) and observed no differences in exosomal total protein contents compared to the mock group (Fig. S5a). Such ECExos (2,000 particles/cell) induced no effect on the paracellular ZO-1 of normal human recipient BMECs (Fig. S5b and c).

10.1128/mBio.00769-21.6 FIG S5 (a) Quantities of ECExos from mock-, heat-inactivated *R. parkeri*-, or *R. parkeri-*infected HUVECs 72 h p.i. at an MOI of 10 were assessed using the total protein concentration of ECExos (*n* = 4 per group). (b and c) Immunofluorescence staining of tight junctional protein ZO-1 (red) and relative fluorescent intensities of paracellular ZO-1. Statistical significance was determined using one-way analysis of variance. Scale bar indicates 20 μm. *, *P* < 0.05. Download FIG S5, JPG file, 0.4 MB. Copyright © 2021 Liu et al. 2021 Liu et al. https://creativecommons.org/licenses/by/4.0/ This content is distributed under the terms of the Creative Commons Attribution 4.0 International license.

These data suggest that *R*-ECExos can induce normal human recipient EC barrier dysfunction in an exosomal RNA cargo-dependent manner.

### *R. parkeri* infection upregulates exosomal mir-23a and mir-30b.

**FIG 5:**
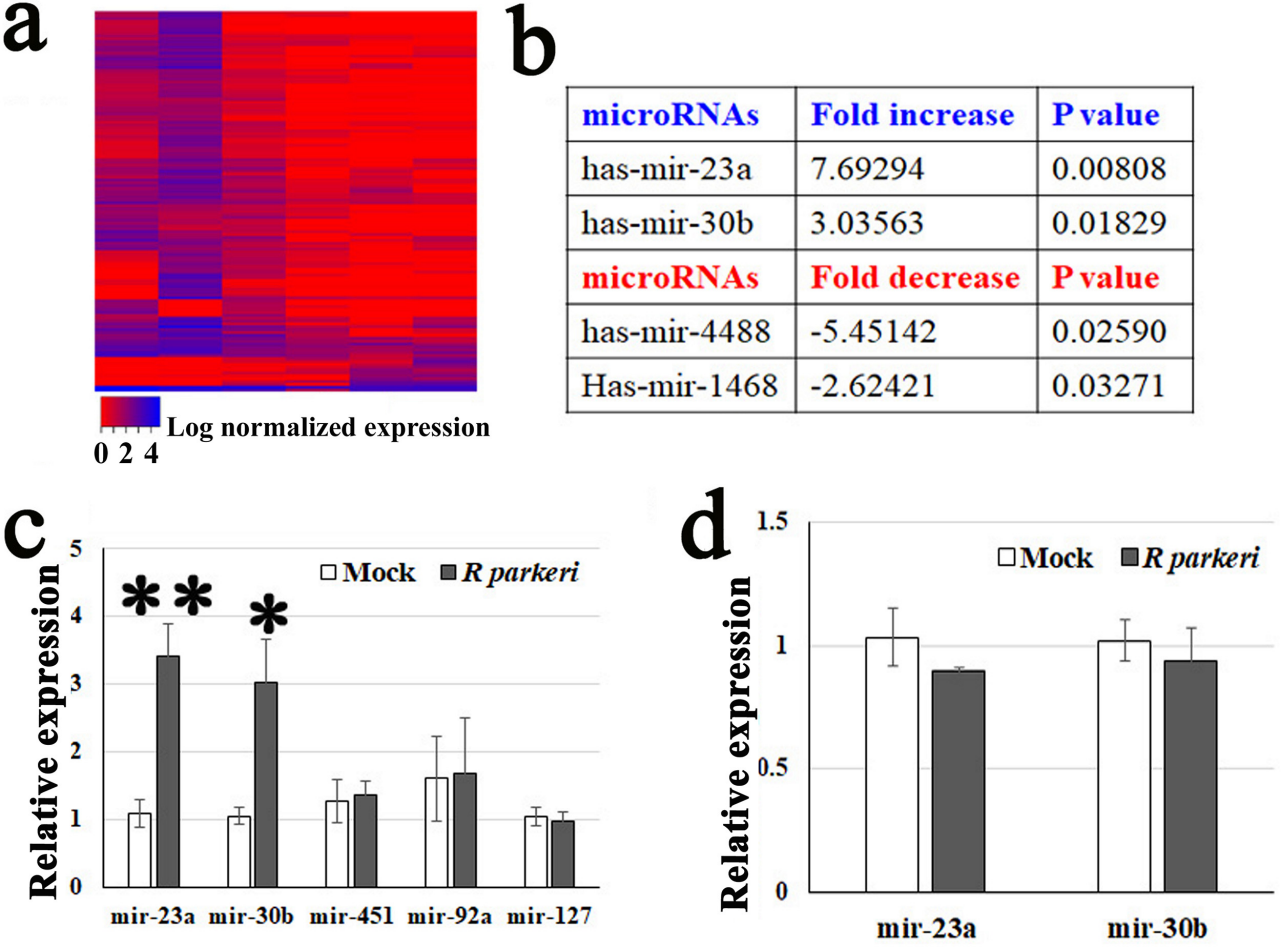
Rickettsial infection alters microRNA expression in ECExos. (a) Heat map clustering of microRNAs in normal ECExos versus *R*-ECExos (*n* = 3). (b) microRNA expression in *R*-ECExos versus normal ECExos (*n* = 3). (c) Stem-loop RT-qPCR analysis of microRNAs obtained from normal ECExos (mock) and *R*-ECExos (rickettsial). **, *P* < 0.01; *, *P* < 0.05. (d) Stem-loop RT-qPCR analysis of microRNAs obtained from normal (mock) and rickettsia-infected donor HUVECs. Statistical significance was determined using one-way analysis of variance.

EV RNA cargo mostly consists of sncRNAs, mainly microRNAs and tRNA-derived fragments (61, 77, 78). A growing number of reports have established that many effects of EVs are mediated by microRNAs (52, 55–60, 63). We characterized the exosomal microRNA cargo using next-generation sequencing (Fig. 5a). RNAs were isolated from Exos released from HUVECs infected with *R. parkeri* (at an MOI of 10) for 72 h or mock infected. *R. parkeri* is a biosafety level 2 (BSL2) pathogen, which lends itself to mechanistic studies. There were no differences in total sncRNAs (<150 nucleotides) per Exo obtained from normal ECExos compared with *R*-ECExos. Seventy-two hours after *R. parkeri* infection, mir-23a and mir-30b exhibited the greatest induction of expression in *R*-ECExos, reaching 7.69-fold and 3.04-fold increases compared to controls, respectively (Fig. 5b).

We next validated the enhanced expression of mir-23a and mir-30b in Exos using stem-loop RT-qPCR, which is a common method for detecting sncRNAs in EVs (52, 61, 97, 98). In Fig. 5c, exosomal mir-23a was upregulated after rickettsial infection with a 3-fold increase in expression compared to the mock group (*P* < 0.01). Similarly, mir-30b had a nearly 3-fold increase (*P* < 0.05). However, the levels of mir-127 (99), mir-451, and mir-92a were stable between mock ECExos and *R*-ECExos (Fig. 5c). Furthermore, we did not detect different levels of these miRNAs in cell samples between groups (Fig. 5d). mir-23a (100–103) and mir-30b (103, 104) have been documented to target endothelial barrier functions. Analysis of the interactions among mir-23a and mir-30b and potential mRNA targets (Tables S1 and S2) suggests putative mRNA candidates potentially associated with vascular endothelial barrier functions.

10.1128/mBio.00769-21.8 TABLE S1 GO enrichment analysis of mir-23a-3p targeted genes. ^a^Count is the number of genes in the user-provided lists with membership in the given ontology term. Log10(P) is the *P* value in log base 10. Download Table S1, DOCX file, 0.01 MB. Copyright © 2021 Liu et al. 2021 Liu et al. https://creativecommons.org/licenses/by/4.0/ This content is distributed under the terms of the Creative Commons Attribution 4.0 International license.

10.1128/mBio.00769-21.9 TABLE S2 GO enrichment analysis of mir-30b-5p targeted genes. ^a^Count is the number of genes in the user-provided lists with membership in the given ontology term. Log10(P) is the *P* value in log base 10. Download Table S2, DOCX file, 0.01 MB. Copyright © 2021 Liu et al. 2021 Liu et al. https://creativecommons.org/licenses/by/4.0/ This content is distributed under the terms of the Creative Commons Attribution 4.0 International license.

Collectively, our data suggest that mir-30b and mir-23a are selectively sorted into *R*-ECExos following *R. parkeri* infection.

We have performed an additional experiment to examine mouse mir-23a and mouse mir-30b in mouse plsExos using stem-loop RT-qPCR. As shown in Fig. S6, levels of mouse plsExo mir-23a (*P* = 0.413) and mir-30b (*P* = 0.237) increased after *R. parkeri* infection, but the results were not statistically significant.

10.1128/mBio.00769-21.7 FIG S6 Stem-loop RT-qPCR analysis of microRNA expression in plsExos. microRNA expression in normal plsExos versus *R*-plsExos (*n* = 6). *R*-plsExos were isolated from mice on day 4 after infection with 2 LD_50_ of *R. parkeri* given by the intravenous route. Statistical significance was determined using one-way analysis of variance. No statistical differences were found using a *P* value of <0.05. Download FIG S6, JPG file, 0.05 MB. Copyright © 2021 Liu et al. 2021 Liu et al. https://creativecommons.org/licenses/by/4.0/ This content is distributed under the terms of the Creative Commons Attribution 4.0 International license.

## DISCUSSION

ECs are the primary mammalian host target cells of SFR infection (2, 5, 6). The most prominent pathophysiological effect during SFR infections is increased microvascular permeability, followed by vasogenic cerebral edema and noncardiogenic pulmonary edema with potentially fatal outcomes (2, 5). Cellular and molecular mechanisms underlying endothelial barrier dysfunction in rickettsiosis remain largely unknown (7–9). The novel findings in the present study are that *R. australis* or *R. parkeri* infection increases the release of heterogeneous plsExos, but endothelial Exo size, morphology, and production are not significantly altered following infection. Mouse *R*-plsExos induced dysfunction of normal mouse recipient BMECs in a dose-dependent manner, and human *R*-ECExos induced dysfunction of normal human recipient BMECs in an exosomal RNA cargo-dependent manner. Next-generation sequencing and stem-loop RT-qPCR analyses suggested that mir-23a and mir-30b are selectively sorted into *R*-ECExos after *R. parkeri* infection. To our knowledge, this is the first report involving EVs in obligately intracellular bacterial infections.

Exos are in a size range similar to that of viruses (33, 94) and contain many types of biomolecules, including proteins and nucleic acids, which contribute to diseases pathogenesis. Exos are being actively investigated in cancers, as biomarkers, and as potential therapeutics (33–38, 68, 70). Exos have been studied in the context of different infections (33–38, 70, 71). During infection, EVs released from the host can be derived from the pathogen or the host. It has been reported that pathogens can utilize different mechanisms to hijack host Exos to maintain their survival and increase their pathogenicity (33). Mycobacterium tuberculosis releases lipoarabinomannan into Exos to decrease the interferon response of the recipient macrophage (33). Exosomal gp63 from *Leishmania* has been shown to downregulate proinflammatory genes in dendritic cells and macrophages (105). Exos released from Leishmania donovani-infected macrophages block the formation of microRNA-122 in recipient hepatocytes, resulting in a higher parasite burden (106). However, most enveloped virions are the same size as Exos, and major exosomal surface markers CD63 and CD81 are enriched in enveloped viruses (54). Such similarities make the separation of virions and Exos in infected samples particularly challenging (54). Rickettsiae are strictly intracellular bacteria that are about 2.0 μm in length (22, 107, 108). We succeeded in isolating and purifying bacterium-free plsExos and ECExos from rickettsia-infected mouse plasma and cell culture media, respectively, taking advantage of SEC technology, which now serves as the technical foundation for studying the potential role of Exos in the pathogenesis of rickettsiosis.

Differential centrifugation has been employed for Exo isolation for many years, but the technique suffers from aggregation and decreased integrity of Exos (38, 53, 64–66). Recently, single-step SEC was employed successfully for Exo purification, with improved integrity, yield, and no aggregation (38, 53, 64–66). In the present study, using SEC technology, we have successfully isolated Exos from plasma and culture media in BSL2/3 facilities and validated Exo quality using multiple EV-specific assays (Fig. 1) demonstrating size, purity, and morphological integrity without aggregation. Exo size and morphology were not significantly changed after rickettsial infection (Fig. 1). Generation of plsExos was significantly upregulated after *R. australis* or *R. parkeri* infection, while no difference was detected in plasma protein concentrations. However, our *in vitro* endothelial *R. parkeri* infection model demonstrated no significant difference in exosomal generation between normal and rickettsia-infected ECs. Circulating Exos have been identified as heterogeneous and derived from multiple different types of cells, including ECs, epithelial cells, leukocytes, erythrocytes, and platelets (90). Our data suggest that ECExo size, morphology, and production were not significantly altered after infection. However, quantitative information regarding the production of SFG rickettsial infection-induced cell-type Exos *in vivo* remains elusive and requires further research.

Research on most species of lethal human SFG rickettsial infections, including R. rickettsii (3, 4), R. conorii (9), and *R. australis* (10), is restricted to BSL3 facilities. One key experimental method in our study was to employ novel SEC technology to isolate high-quality Exos from plasma and culture media. Vascular ECs are exceedingly thin (109). Preliminary experiments revealed that we obtained smaller amounts of Exo particles from ECs cultured in the same-size culture vessel at the same confluence for the same duration compared with other cell types. It is practical and feasible to isolate endothelial Exos from media using SEC on a relatively larger scale following infection with *R. parkeri* (5–7), which requires a lower biocontainment level than *R. australis*. *R. parkeri*, the cause of *R. parkeri* rickettsiosis (8), is proposed as an experimental pathogen for studying the pathogenesis of SFG rickettsiosis and can be experimentally handled at BSL2 (6, 95). In a murine model of intravenous inoculation of *R. parkeri* (Atlantic Rainforest strain) using C3H/HeN mice, signs of illness begin on day 3 and fatalities occur on day 6 p.i. (95), during which time microvascular damage is detected in multiple organs (95); this is similar to the pathology observed in our murine model of *R. australis* infection (85, 87). However, differences among various pathogen species must be considered in future mechanistic studies. Furthermore, current technological hurdles hamper us from isolating ECExos from plasma on a large scale for downstream experiments. To collect sufficient ECExos using SEC technology for mechanistic studies, culturing cells in media is the sole practical source for ECExo isolation. To investigate the functional role of ECExos in *R. parkeri* infection, we used HUVECs as the source of Exo-producing cells and BMECs as the recipient cells. Research to develop feasible cell-type Exo isolation using the same pathogens in the same host models is warranted for future mechanistic studies.

Exos can induce functional responses using multiple mechanisms, including releasing bioactive components after internalization (67, 68). In infectious disease biology, EVs from infected donor cells are associated with virulence of the pathogens in recipient cells (33–38, 69–71). In the present study, both *R*-plsExos and *R*-ECExos weakened the barrier function of the normal ECs. Concomitantly, human *R*-ECExos induced disruption of the tight junctional protein ZO-1 in recipient human BMECs in an exosomal RNA-dependent manner. However, the underlying mechanism remains unclear.

The discovery of extracellular sncRNAs in the blood, despite the abundant presence of RNases, led to the proposal of a scenario in which sncRNAs are encapsulated in EVs (55, 72–74) or in the form of circulating ribonucleoproteins (75, 76). EV-enclosed mRNAs are mostly fragmented, and extracellular RNAs are enriched in sncRNAs (77). Despite a previous report that the average copy number of miRNAs in each EV is low (110), accumulating evidence suggests a critical function of EV-containing miRNAs. EV RNA cargo mostly consists of sncRNAs (77). A growing number of reports have established that many, if not all, of the effects of EVs are mediated by microRNAs (52, 55–60, 63), which remain functional to regulate cellular behaviors of the recipient cell (79). Exosomal microRNAs are of particular interest due to their participation in posttranslational regulation of gene expression. A single microRNA can regulate many target genes to affect biological function (111). Recent studies provide evidence that microRNAs are selectively sorted into EVs, independent of their cellular levels (52, 55–62). We observed no significant differences in total sncRNAs (<150 nucleotides) per Exo between normal ECExo and *R*-ECExo. Seventy-two hours after *R. parkeri* infection, expression levels of mir-23a and mir-30b were remarkably upregulated in *R*-ECExos, but no change was observed in the mock-infected cells. These data suggest that mir-23a and mir-30b are selectively sorted into *R*-ECExos during *R. parkeri* infection. The underlying mechanism is yet to be elucidated.

Analysis of the interactions among enriched exosomal microRNAs and potential mRNA targets will provide putative mRNA candidates for future studies. Given that the molecular and functional effects of mir-23a (100–103) and mir-30b (103, 104) have been documented to target endothelial barrier functions, further research into the selective sorting mechanism(s) and functional roles of exosomal mir-23a and mir-30b may provide new insights into the pathogeneses of SFR. Furthermore, additional research may validate specific exosomal microRNAs as impactful druggable targets for the prevention and treatment of fatal human diseases caused by *Rickettsia* and other pathogens.

## MATERIALS AND METHODS

### Mouse models of *R. australis* and *R. parkeri* infections.

All animal experiments were performed according to protocols approved by the Institutional Animal Care and Use Committee of the University of Texas Medical Branch (UTMB). C57BL/6J mice were obtained from the Jackson Laboratory (Bar Harbor, ME). Mice used for *R. australis* infections were 8- to 12-week-old males. C57BL/6J mice are highly susceptible to *R. australis*, becoming ill on day 3 p.i. and succumbing to infection on day 6. Therefore, this organism was chosen as the SFG rickettsial agent for the *in vivo* studies (10). Male C57BL/6 mice infected with *R. australis* are an established animal model of human SFG rickettsiosis because the pathology involves disseminated endothelial infection and pathological lesions, including vasculitis in multiple organs, similar to what is observed in human SFG rickettsiosis (10, 87). However, use of *R. australis* requires BSL3 containment. An ordinarily lethal dose of 2 LD_50_ of *R. australis* (the LD_50_ is 1 × 10^6^ PFU) was injected through the tail vein (87), and whole-blood samples were collected on day 4 p.i. for plasma isolation.

*R. parkeri* is proposed as an experimental pathogen for studying the pathogenesis of SFG rickettsiosis and is similar to *R. australis* (10, 84, 86, 87), but it can be experimentally handled using BSL2 containment (6, 95). For isolation of endothelial Exos from infected media on a large scale, it is more practical and feasible to use *R. parkeri*. In a murine model of intravenous inoculation of *R. parkeri* (Atlantic Rainforest strain) using C3H/HeN mice, signs of illness began on day 3 and fatalities occurred on day 6 p.i. (95), during which time microvascular damage was detected in multiple organs (95), which is similar to the pathology observed in the murine model of *R. australis* (85, 87). C3H/HeJ mice (8- to 12-week-old males) were obtained from the Jackson Laboratory. An ordinarily lethal dose of 2 LD_50_ of *R. parkeri* (Atlantic Rainforest strain) was injected through the tail vein (87), and blood samples were collected on day 4 p.i. for plasma isolation.

### NTA.

To determine the size and concentration of EVs, nanoparticle tracking analysis (NTA) was performed at the Nanomedicines Characterization Core Facility (University of North Carolina, Chapel Hill, NC). Briefly, isolated Exo samples were diluted to a concentration of 5 × 10^9^ to 1 × 10^11^ particles/ml in filtered PBS. The samples were then processed on a NanoSight NS500 (NanoSight, Malvern Instruments, Westborough, MA) to capture particles moving by way of Brownian motion (camera type, scientific complementary metal oxide semiconductor [sCMOS]; camera level, 16; detection threshold, 5). The hydrodynamic diameters were calculated using the Stokes-Einstein equation. The 100-nm standard particles and the diluent PBS alone were used for reference.

### MicroRNA quantification in HUVEC Exos.

As previously reported (52), RNAs were extracted from purified HUVEC Exos. Small RNAs (6 to 150 nucleotides) and microRNA fractions (10 to 40 nucleotides) were quantified using high-resolution small RNA analysis (2100 Bioanalyzer system; Agilent, Santa Clara, CA) at the Biopolymer Facility (Harvard Medical School, Cambridge, MA). To determine the concentration of small RNAs and microRNAs per Exo, the quantified sncRNA/microRNA value was normalized to the Exo count, which was evaluated using NTA.

### Bioinformatic analysis of sequencing data.

Sequencing was done using an Illumina NextSeq as single-end 75-bp reads generating between 4.8 and 40.9 million reads per sample. Quality control of the samples was performed using Qiagen CLC Genomics Workbench 20.0. Raw sequencing reads were trimmed to remove Qiagen 3′-AACTGTAGGCACCATCAAT and 5′-GTTCAGAGTTCTACAGTCCGACGATC adapters, as well as filtered based on initial quality assessment. Reads dominated by low-quality base calls, and longer than 55 nucleotides, were excluded from the downstream analyses. Filtered data underwent further transcriptome sequencing (RNA-Seq) analysis using the CLC Genomics Workbench 20.0 RNA-Seq Analysis 2.2 module with RNAcentral noncoding human RNA (112, 113) (downloaded 16 April 2020), miRbase 22.1, and the ENSMBL GRCh38 noncoding RNA gene collection (114) (downloaded 20 November 2019). Differential expression analysis was performed using the Differential Expression in Two Groups 1.1 module. The differential expression module uses multifactorial statistics based on a negative binomial generalized linear model (GLM) to correct for differences in library size between the samples and the effects of confounding factors. The Wald test was used to compare the expression of noncoding RNA between the groups.

### Atomic force microscopy (AFM).

The purified and concentrated EV samples were diluted at 1:10, 1:100, and 1:1,000 with molecular-grade water. Glass coverslips were cleaned three times with ethanol and acetone and then three times with molecular-grade water. Each coverslip was correctly labeled, placed in the hood to dry under laminar flow for 1 h, and subjected to coating with the diluted EV samples on the designated area for 30 min. EV samples were washed away gently with molecular-grade water, and the coverslip was dried for 1 h.

The coverslip coated with EV samples was examined using an AFM (CoreAFM; Nanosurf AG, Liestal, Switzerland) using contact mode in the air. A PPP-FMR-50 probe (0.5 to 9.5 N/m, 225 μm in length, and 28 μm in width; Nanosensors, Neuchatel, Switzerland) was used. The parameters of the cantilever were calibrated using the default script from the CoreAFM program using the method of Sader et al. (115). The cantilever was approached to the sample under the set point of 20 nN (115), and topography scanning was done using the following parameters: 256 points per line and 1.5 s per line in a 5-μm by 5-μm image.

### Rickettsiae, cell culture, and rickettsial infections.

*R. australis* (Cutlack strain) (87) and *R. parkeri* (Atlantic Rainforest strain) (95) were prepared as described previously. Uninfected Vero cells were processed as mock-infected control material using the same procedure. All biosafety level (BSL)2/3 and animal BSL2/3 experiments were performed in CDC-certified facilities in the Galveston National Laboratory at UTMB, Galveston, TX, using established procedures.

A standard protocol to isolate brain microvascular endothelial cells (BMECs) from C57BL/6 and C3H/HeN mice (116) was used. Human umbilical vein endothelial cells (HUVECs; Cell Applications, Inc.) or BMECs were cultivated in 5% CO_2_ at 37°C on type I rat tail collagen-coated round glass coverslips (12-mm diameter; Ted Pella, Redding, CA) until 90% confluence was observed. HUVECs were infected with *R. parkeri* at an MOI of 10. Uninfected ECs were used as mock controls and were subjected to the same procedure. All experiments were performed in triplicate. Normal mouse or rabbit IgGs were used as negative controls.

### ECExo and plsExo isolation, concentration, and permeabilization.

**(i) ECExo isolation and concentration.** Donor HUVECs in T75 flasks were infected using *R. parkeri* at an MOI of 10 or were mock infected for 72 h, and 11 ml of medium was collected. The media were passed through 0.2-μm syringe filters twice. Following the manufacturer’s instructions, 10 ml of filtered medium was subjected to the qEV10 column (Izon, New Zealand) for SEC isolation. Four fractions (the number 7 to 10 fractions) were collected as the Exo-enriched fractions, which were concentrated using 100,000-molecular-weight-cutoff (MWCO) polyethersulfone (PES) Vivaspin centrifugal filters (Thermo Fisher Scientific). Exo samples (in 200 μl of PBS) were stored at −80°C prior to use in downstream assays.

**(ii) plsExo isolation and concentration.** For plasma isolation, blood samples were collected in anticoagulation tubes 4 days following infection with *R. australis*, *R. parkeri*, or saline (mock infected). The plasma sample (200 μl) was passed through 0.2-μm syringe filters twice. Following the manufacturer’s instructions, filtered plasma was placed onto the qEVoriginal column (Izon, New Zealand) for SEC isolation. The number 7 to 9 fractions were collected as the Exo-enriched fractions, which were concentrated using 100,000-MWCO PES Vivaspin centrifugal filters (Thermo Fisher Scientific). Exo samples (in 200 μl of PBS) were stored at −80°C prior to use in downstream assays. Following the manufacturer’s instructions, three fractions (the number 13 to 15 fractions) from the qEVoriginal column isolation were collected as the Exo-free, high-protein plasma fractions, which were concentrated using centrifugal filters and used in Western immunoblotting as a control.

For saponin-assisted active exosomal permeabilization pretreatment (81–83) of Exos using RNase, Exo samples (1 × 10^9^ particles/ml) and RNase (20 μg/ml) (Thermo Fisher Scientific) were incubated with 0.1 mg/ml of saponin (Thermo Fisher Scientific) at room temperature for 15 min. After being rinsed using PBS, Exo samples were concentrated using 100,000-MWCO PES Vivaspin centrifugal filters.

### Distribution of Exos *in vivo* and *in vitro*.

Using a published approach, recipient cell uptake of Exos was assessed *in vivo* and *in vitro* (90). Briefly, following incubation using materials from the PKH26 red fluorescent cell linker kit (Millipore Sigma, St. Louis, MO), the PKH26 red-prelabeled Exos were washed three times with PBS prior to ultracentrifugation at 100,000 × *g* for 20 min at 4°C using a Beckman L7-80 and SW41 rotor (Beckman Coulter, Indianapolis, IN) to remove unbound stain. PBS without Exos was processed using the same steps as the mock PKH26-labeled tracer. A single injection of PKH26-labeled exosomes (about 1 × 10^11^ particles in 100 μl of PBS) via the tail vein of a normal mouse was done to observe the distribution of Exos in the lungs, liver, and brain 6 h after injection. Immunofluorescence staining was done using frozen sections with rabbit antibodies to CD31. For the *in vitro* assessment, living BMECs were exposed to PKH26-prelabeled ECExos (2,000 particles/cell) in the culture media of normal human BMECs. After 2 h, cells were fixed and immediately subjected to fluorescence microscopy. All solutions of PKH26-labeled ECExos were filtered with a 0.2-μm filter. Fluorescent images were analyzed using Olympus BX51 epifluorescence and a Nikon A1R MP ECLIPSE T*i* confocal microscope with *NIS*-Elements imaging software version 4.50.00 (Nikon, Tokyo, Japan).

### Stem-loop real-time PCR.

Total RNA was extracted from EVs by using TRIzol (Invitrogen). An exogenous synthetic microRNA, namely, cel-mir-39, was diluted in TRIzol before extraction to act as a normalizer. The concentration of total RNA was measured by NanoDrop (ND-2000). Primers for cel-mir-39, has-mir-23a, has-mir-30b, has-mir-92a, has-mir-451, has-mir-127, mmu-mir-23a, mmu-mir-30b, mmu-mir-92a, and mmu-mir-127 for TaqMan microRNA assay INV SM 10 were purchased from Thermo Fisher Scientific. A TaqMan microRNA reverse transcription kit (Applied Biosystems) was used for reverse transcription reactions. The 15-μl RT reaction mixtures contained 5 ng of total RNA template, 3 μl of RT primer (5×), 0.15 μl of deoxynucleoside triphosphate (dNTPs; 100 mM), 1 μl of MultiScribe reverse transcriptase (50 U/μl), 1.5 μl of reverse transcription buffer (10×), 0.19 μl of RNase inhibitor (20 U/μl), and 4.16 μl of nuclease-free water. Reverse transcription conditions were 16°C for 30 min, 42°C for 30 min, and 85°C for 5 min. For PCR amplification, the 10-μl PCRs included 0.7 μl of cDNA template acquired as described above, 0.5 μl of TaqMan small RNA assay mix (20×), 5 μl of PCR master mix, and 3.8 μl of nuclease-free water. qPCR conditions were 50°C for 2 min and 95°C for 30 s, followed by 40 cycles of 95°C for 5 s and 65°C for 30 s. The relative expression of each miRNA was expressed as 2^−ΔΔ^*^CT^* by the CFX Connect real-time system (Bio-Rad, Hercules, CA).

### Statistics.

Statistical significance was determined using Student’s *t* test or one-way analysis of variance. Results were regarded as significant if two-tailed *P* values were <0.05. All data are expressed as means ± standard errors of the means.

10.1128/mBio.00769-21.1 TEXT S1 Supplemental Materials and Methods. Download Text S1, DOCX file, 0.03 MB. Copyright © 2021 Liu et al. 2021 Liu et al. https://creativecommons.org/licenses/by/4.0/ This content is distributed under the terms of the Creative Commons Attribution 4.0 International license.
