# Supplementary figures and images for: Endothelial Exosome Plays a Functional Role during Rickettsial Infection
Source: mBio. 2021 May 11;12(3):e00769-21. doi: 10.1128/mBio.00769-21 (PMC8262936; doi:10.1128/mBio.00769-21)

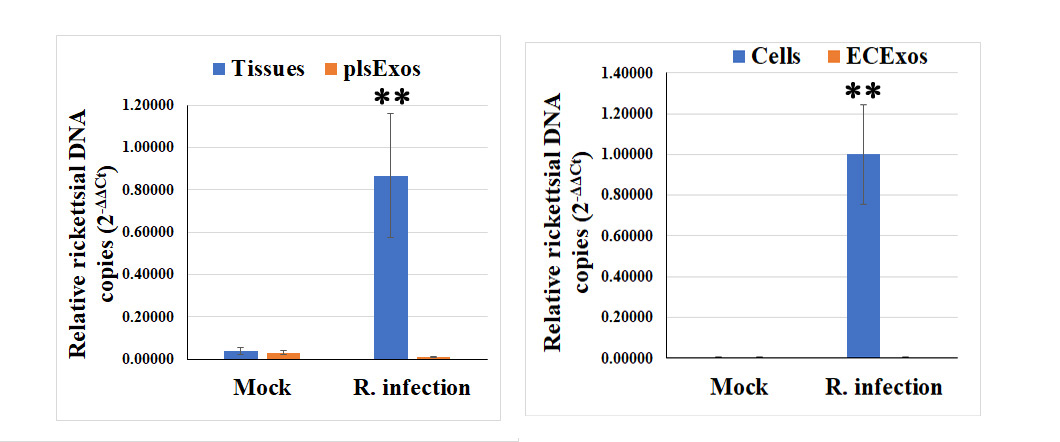

Supplement: FIG S1 [file mbio.00769-21-sf001.jpg]

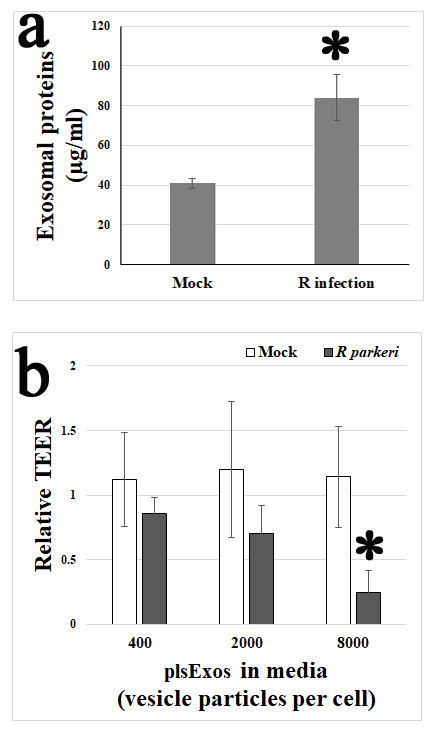

Supplement: FIG S2 [file mbio.00769-21-sf002.jpg]

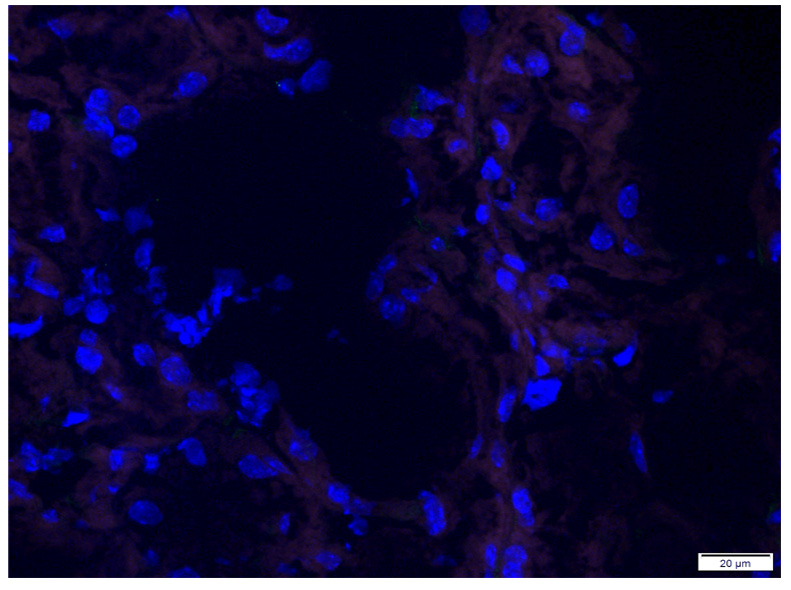

Supplement: FIG S3 [file mbio.00769-21-sf003.jpg]

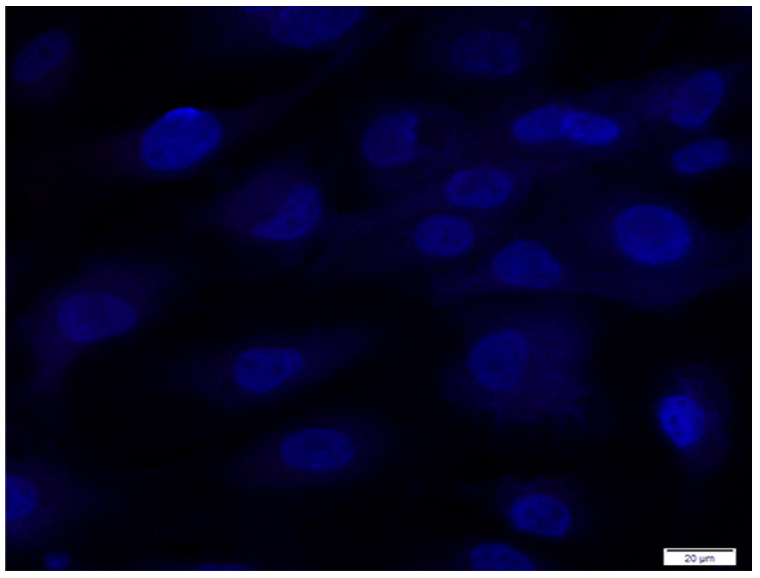

Supplement: FIG S4 [file mbio.00769-21-sf004.jpg]

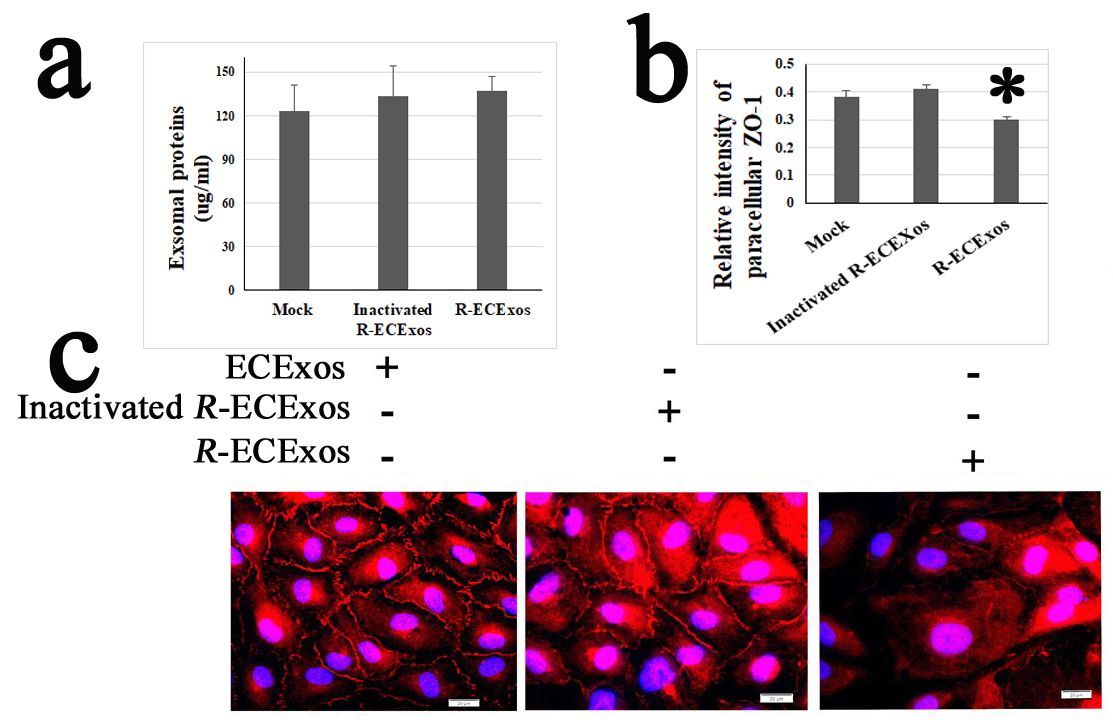

Supplement: FIG S5 [file mbio.00769-21-sf005.jpg]

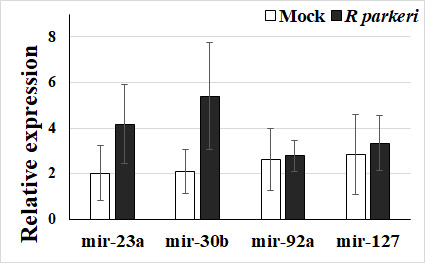

Supplement: FIG S6 [file mbio.00769-21-sf006.jpg]
