# Supplementary material for: Endothelial Exosome Plays a Functional Role during Rickettsial Infection
Source: mBio. 2021 May 11;12(3):e00769-21. doi: 10.1128/mBio.00769-21 (PMC8262936; doi:10.1128/mBio.00769-21)
Supplement: TABLE S1 [file mbio.00769-21-st001.docx]

| **GO** | **Category** | **Description** | **Count(%)^a^** | **Log10(P)** |
| --- | --- | --- | --- | --- |
| GO:0016301 | GO Molecular Functions | kinase activity | 7.73 | -21.64 |
| GO:0001568 | GO Biological Processes | blood vessel development | 7.05 | -15.43 |
| GO:0045664 | GO Biological Processes | regulation of neuron differentiation | 6.68 | -17.54 |
| GO:0003682 | GO Molecular Functions | chromatin binding | 6.23 | -17.83 |
| GO:0071363 | GO Biological Processes | cellular response to growth factor stimulus | 6.15 | -12.63 |
| GO:1905114 | GO Biological Processes | cell surface receptor signaling pathway involved in cell-cell signaling | 5.93 | -14.51 |
| GO:0045596 | GO Biological Processes | negative regulation of cell differentiation | 5.93 | -9.84 |
| GO:0001228 | GO Molecular Functions | DNA-binding transcription activator activity, RNA polymerase II-specific | 5.78 | -18.33 |
| GO:0032870 | GO Biological Processes | cellular response to hormone stimulus | 5.78 | -11.73 |
| GO:0019904 | GO Molecular Functions | protein domain specific binding | 5.63 | -9.89 |
| GO:0001501 | GO Biological Processes | skeletal system development | 5.55 | -16.61 |
| GO:0048589 | GO Biological Processes | developmental growth | 5.55 | -10.78 |
| GO:0045860 | GO Biological Processes | positive regulation of protein kinase activity | 5.18 | -13 |
| GO:0007610 | GO Biological Processes | behavior | 5.18 | -10.58 |
| GO:0048568 | GO Biological Processes | embryonic organ development | 4.65 | -13.11 |
| GO:0050808 | GO Biological Processes | synapse organization | 4.2 | -10.9 |
| GO:0098978 | GO Cellular Components | glutamatergic synapse | 4.05 | -13.07 |
| GO:0030099 | GO Biological Processes | myeloid cell differentiation | 4.05 | -9.93 |
| GO:0060485 | GO Biological Processes | mesenchyme development | 3.6 | -13.36 |
| GO:0001655 | GO Biological Processes | urogenital system development | 3.6 | -11.19 |
