# Supplementary material for: Endothelial Exosome Plays a Functional Role during Rickettsial Infection
Source: mBio. 2021 May 11;12(3):e00769-21. doi: 10.1128/mBio.00769-21 (PMC8262936; doi:10.1128/mBio.00769-21)
Supplement: TABLE S2 [file mbio.00769-21-st002.docx]

| **GO** | **Category** | **Description** | **Count(%)^a^** | **Log10(P)** |
| --- | --- | --- | --- | --- |
| GO:0120039 | GO Biological Processes | plasma membrane bounded cell projection morphogenesis | 7.14 | -24.29 |
| GO:1990234 | GO Cellular Components | transferase complex | 6.82 | -17.51 |
| GO:0007420 | GO Biological Processes | brain development | 6.57 | -16.1 |
| GO:0016301 | GO Molecular Functions | kinase activity | 6.38 | -15.25 |
| GO:0009792 | GO Biological Processes | embryo development ending in birth or egg hatching | 6.12 | -16.88 |
| GO:0003006 | GO Biological Processes | developmental process involved in reproduction | 5.93 | -13.93 |
| GO:0019904 | GO Molecular Functions | protein domain specific binding | 5.8 | -12.72 |
| GO:0030424 | GO Cellular Components | axon | 5.74 | -14.98 |
| GO:0032870 | GO Biological Processes | cellular response to hormone stimulus | 5.68 | -13.12 |
| GO:0001228 | GO Molecular Functions | DNA-binding transcription activator activity, RNA polymerase II-specific | 5.61 | -20.22 |
| GO:0048589 | GO Biological Processes | developmental growth | 5.61 | -13.06 |
| GO:0061061 | GO Biological Processes | muscle structure development | 5.61 | -12.59 |
| GO:1905114 | GO Biological Processes | cell surface receptor signaling pathway involved in cell-cell signaling | 5.36 | -12.97 |
| GO:0016569 | GO Biological Processes | covalent chromatin modification | 4.78 | -14.95 |
| GO:0098793 | GO Cellular Components | presynapse | 4.78 | -13.06 |
| GO:0007264 | GO Biological Processes | small GTPase mediated signal transduction | 4.66 | -12.77 |
| GO:0098978 | GO Cellular Components | glutamatergic synapse | 4.4 | -18.69 |
| GO:0009896 | GO Biological Processes | positive regulation of catabolic process | 4.4 | -13.43 |
| GO:1903311 | GO Biological Processes | regulation of mRNA metabolic process | 3.64 | -12.75 |
| GO:1990823 | GO Biological Processes | response to leukemia inhibitory factor | 1.79 | -12.52 |
