# Supplementary material for: Endothelial Exosome Plays a Functional Role during Rickettsial Infection
Source: mBio. 2021 May 11;12(3):e00769-21. doi: 10.1128/mBio.00769-21 (PMC8262936; doi:10.1128/mBio.00769-21)
Supplement: TEXT S1 [file mbio.00769-21-s0001.docx]

**Supplemental Materials**

**Materials and Methods**

**Antibodies and other reagents**

Anti-AnnexinA2 mouse monoclonal antibody (mAb) (clone 666316) was purchased from R&D Systems (Minneapolis, MN) via Thermo Fisher Scientific (Rockford, IL). Anti-VE-cadherin, anti-CD31, anti-CD45, and anti-CD63 rabbit antibodies were purchased from Abclonal (Woburn, MA). Anti-flotillin-1 and anti-calnexin mouse antibodies were purchased from BD Transduction Laboratories (San Jose, CA). Anti-TSG101 rabbit antibody was purchased from Novus Biologicals (Centennial, CO). Anti-albumin rabbit antibody was purchased from Cell Signaling Technology (Danvers, MA). Anti-ZO-1 rabbit antibody, AlexaFluor 488-conjugated goat anti-mouse IgG, AlexaFluor 594-conjugated goat anti-rabbit IgG, and DAPI were purchased from Invitrogen (Carlsbad, CA). Normal mouse and rabbit IgGs were purchased from Agilent (Santa Clara, CA). Endothelial Cell Growth Medium and fetal bovine sera were obtained from Cell Applications, Inc. (San Diego, CA). MicroBCA protein assay kit and Pierce^TM^ BCA kit were purchased from Thermo Fisher Scientific. To ensure that there were adequate Exo samples for downstream experiments, Exo samples had to be diluted at 1:15 for the BCA protein assay. Preliminary experiments showed that the sensitivity of the Pierce^TM^ BCA kit was lower in the diluted Exo samples. Therefore, as other groups reported (1, 2), the MicroBCA assay was utilized to measure exosomal protein concentrations. Absorbance was detected using a Bio-Tek Microplate reader at 562nm. Unless otherwise indicated, all reagents were purchased from Thermo Fisher Scientific.

Heat-inactivated *R. parkeri* were obtained by heating organisms at 60°C for 30 min (3).

**Transmission electron microscopy** (**TEM)**

Negative staining was used to visualized EVs using TEM. A TEM grid was coated with 0.01% bovine serum albumin (BSA) for 30 seconds. Then the BSA was drawn off the TEM grid and 10 ul of EV sample was immediately added and left to absorb for one minute. The grid was then washed with 1mM ethylenediaminetetraacetic acid (EDTA) and stained with 10 ul 0.5% uranyl acetate, which was drawn off from the edge of the grid after one minute. The TEM grid was dried under a heat lamp for three min prior to observation under TEM. All the solutions were filtered using 0.2µm syringe filters.

**Western immunoblotting**

For western immunoblotting, equal amounts of soluble protein were subjected to 10% SDS–polyacrylamide gel electrophoresis (SDS-PAGE). Proteins were transferred onto a polyvinylidene difluoride membrane and then incubated with primary antibody (1:1,000 for anti-Flotillin-1, albumin, AnnexinA2, CD63, TSG101, or calnexin-1 antibodies) at 4℃ overnight, followed by incubation with a secondary antibody at 1:10,000 for 2 hrs. A goat anti-mouse or anti-rabbit IgG and IgM (H+L)-HRP (Thermo Fisher Scientific) were used as the secondary antibodies. Blots were visualized using the Pierce™ ECL Western Blotting Substrate kit (Thermo Fisher Scientific). “Exo-free plasma” samples were loaded at 30 µg in each lane.

**Transendothelial electrical resistance (TEER)**

TEER was measured using a EVOM resistance meter (Millicell ERS-2) (Thermo Fisher Scientific, Rockford, IL), as reported (4). BMEC monolayers were seeded on inserts in 24-well plates (0.4 µm polyester membrane, CoStar, Thermo Fisher Scientific), and TEERS were measured after 72 hrs of treatment. The values are shown as Ω×cm^2^ and normalized by subtracting the background (i.e., TEER from an insert without cells).

**Immunofluorescence (IF)**

For IF staining of CD31 in mouse tissues collected after extensive *in vivo* perfusion, Ultra V Block and normal rabbit serum were employed for the reduction of nonspecific background before CD31 antibody incubation. Frozen sections of 5 µm thickness were incubated with anti-CD31 rabbit polyclonal antibody (1:500) for 2 hrs at room temperature, followed by AlexaFluor 488-conjugated goat anti-rabbit IgG (1:1000) for 30 min at room temperature. For IF of ZO-1 in BMECs, cells were incubated with anti-ZO-1 rabbit polyclonal antibody (1:500) for 2 hrs, followed by AlexaFluor 594-conjugated goat anti-rabbit IgG (1:1000) for 30 min. Nuclei were counter-stained with DAPI. A rabbit polyclonal IgG (Thermo Fisher) served as a negative control (5) (**Supplemental Fig. 3**). Fluorescent images were analyzed using an Olympus BX51 epifluorescence or Nikon A1R MP ECLIPSE T*i* confocal microscope with *NIS*-Elements imaging software (version 4.50.00) using a final 40x optical zoom.

To score differences in paracellular junctions in fluorescent microscopy images, a published method using ImageJ, an open platform for scientific image analysis, was modified as described (6). In the present study, the ZO-1 signals were located not only at paracellular junctional areas but also in cytosol and nucleic areas. To quantify the relative intensity of ZO-1 at junctional sites, all fluorescent microscope images were processed using the ImageJ/Process/Filters/Top Hat/Reset Radius tool set to the same pixels to filter out fluorescent signals in cytosol and nuclear areas. After processing, the relative intensity of the ZO-1 in each cell was measured using the ImageJ/Analyze/Histogram feature. The results were expressed as the ratio of the total fluorescent signals to cells (nuclei). Twenty microscopic fields were examined for each sample.

**Target predictions and gene enrichment analysis**

We utilized TargetScan (release 7.2) to forecast the genes targeted by mir-23a-3p and mir-30b-5p. The enrichment of the underlying target genes was then analyzed using Metascape (<http://metascape.org/gp/>), which includes gene ontology (GO) biological processes (BPs), GO cellular components (CCs), and GO molecular functions (MFs).

For genes targeted by mir-23a-3p, the top three terms were kinase activity, blood vessel development, and regulation of neuron differentiation (**Supplemental Table 1**). However, for genes targeted by mir-30b-5p, the top terms predominantly involved plasma membrane bounded cell projection morphogenesis, transferase complex, and brain development (**Supplemental Table 2**).

**References**

1. Gaspar LS, Santana MM, Henriques C, Pinto MM, Ribeiro-Rodrigues TM, Girão H, Nobre RJ, Pereira de Almeida L. 2020. Simple and Fast SEC-Based Protocol to Isolate Human Plasma-Derived Extracellular Vesicles for Transcriptional Research. Mol Ther Methods Clin Dev 18:723-737.

2. Gualerzi A, Kooijmans SAA, Niada S, Picciolini S, Brini AT, Camussi G, Bedoni M. 2019. Raman spectroscopy as a quick tool to assess purity of extracellular vesicle preparations and predict their functionality. J Extracell Vesicles 8:1568780.

3. Astrup E, Lekva T, Davì G, Otterdal K, Santilli F, Oie E, Halvorsen B, Damås JK, Raoult D, Vitale G, Olano JP, Ueland T, Aukrust P. 2012. A complex interaction between Rickettsia conorii and Dickkopf-1--potential role in immune evasion mechanisms in endothelial cells. PLoS One 7:e43638.

4. Wilhelm I, Fazakas C, Krizbai IA. 2011. In vitro models of the blood-brain barrier. Acta Neurobiol Exp (Wars) 71:113-28.

5. Liu Y, Xiao J, Zhang B, Shelite TR, Su Z, Chang Q, Judy B, Li X, Drelich A, Bei J, Zhou Y, Zheng J, Jin Y, Rossi SL, Tang SJ, Wakamiya M, Saito T, Ksiazek T, Kaphalia B, Gong B. 2020. Increased talin-vinculin spatial proximities in livers in response to spotted fever group rickettsial and Ebola virus infections. Lab Invest doi:10.1038/s41374-020-0420-9.

6. Terryn C, Sellami M, Fichel C, Diebold MD, Gangloff S, Le Naour R, Polette M, Zahm JM. 2013. Rapid method of quantification of tight-junction organization using image analysis. Cytometry A 83:235-41.
